# *In vitro* and *in vivo* endothelial interactions of *Leptospira* species are markers of virulence

**DOI:** 10.1371/journal.pntd.0013939

**Published:** 2026-01-27

**Authors:** Phillip N. Anderson, Beth L. Hahn, Ger Thao, Molly Sue Johnson, Alexandre Giraud-Gatineau, Yan Gao, Mathieu Picardeau, Jenifer Coburn, Matthew C. Surdel

**Affiliations:** 1 Department of Medicine, Division of Infectious Diseases, Medical College of Wisconsin, Milwaukee, Wisconsin, United States of America; 2 School of Medicine, Medical College of Wisconsin, Milwaukee, Wisconsin, United States of America; 3 Institut Pasteur, Université Paris Cité, Biology of Spirochetes Unit, Paris, France; 4 Division of Biostatistics, Data Science Institute, Medical College of Wisconsin, Milwaukee, Wisconsin, United States of America; 5 Department of Microbiology and Immunology, Medical College of Wisconsin, Milwaukee, Wisconsin, United States of America; Faculty of Medicine and Health Sciences, Universiti Putra Malaysia, MALAYSIA

## Abstract

Leptospirosis is a global zoonotic disease caused by pathogenic species of the genus *Leptospira*. *Leptospira* species are classified into two major clades (pathogenic, P, and saprophytic, S), and four subclades (P1, P2, S1, and S2), with the P1 subclade further divided into high virulence (P1+) and low virulence (P1-) groups. While previous studies have associated P1 + species to greater virulence in the host, phenotypic characterization across clades, particularly regarding dissemination and cell barrier disruption, remains limited. In this study, sixteen strains of pathogenic and saprophytic *Leptospira* representing subclades P1 + , P1-, P2, and S1 were evaluated *in vitro* to assess association with human endothelial cells, disruption of host VE-cadherin localization in adherens junctions, and immune response as measured by cytokine and chemokine release. Our findings indicate that VE-cadherin disruption correlates with P1 + species and the presence of virulence-associated genes. Additionally, bacterial association with host cells correlates with the loss of VE-cadherin localization in adherens junctions. *In vitro Leptospira* interaction with endothelial cells induced production of chemokine and cytokines, most prominent in the P1 + clade and correlating with the presence of virulence-associated genes. Using an *in vivo* murine model of hematogenous dissemination to assess tissue tropism, live *Leptospira* were cultured from relevant tissues of animals inoculated with most of the strains tested and bacterial burdens were quantified to measure adhesion to tissues. Four of the six P1 + strains exhibited significantly higher tissue burdens in kidney, liver, and bladder at one hour post-inoculation compared to other *Leptospira* species. Together, these results suggest that endothelial cell interactions may be a key phenotypic marker for virulence classification in *Leptospira*. Further defining these interactions may therefore provide insights into interventions to combat this potentially fatal disease.

## Author summary

Leptospirosis is a global zoonotic disease and is transmitted to humans through contact with infectious bacteria found in the environment. In this study we tested sixteen strains of *Leptospira*, ranging from non-infectious to highly pathogenic species, in both cell culture and animal models. Disruption of cells and dissemination to tissues were associated with high virulence species. This study contributes to the understanding of *Leptospira*-host interactions, offers insights into the phenotypic diversity of *Leptospira* clades, and identifies phenotypes that can be used as virulence markers in future studies aimed at combatting this potentially fatal zoonotic disease.

## Introduction

Leptospirosis is the most widespread zoonotic disease globally with over one million human cases resulting in approximately 60,000 human deaths annually [1]. Humans typically become infected *via* exposure to pathogenic *Leptospira* present in contaminated soil or water. Rodents and domestic livestock are the main reservoirs for human infection, and shed the bacteria in their urine, thereby contaminating the environment [2]. Natural disasters as well as poor sewage and drainage infrastructure also lead to increased risk for human exposure [3].

*Leptospira* species are highly motile gram-negative spirochetes with periplasmic flagella [4]. They are distinguished from other spirochetes by their hooked ends and ability to survive in a broad range of environmental conditions [5]. Pathogenic strains gain entry through mucosae or breaks in the skin, disseminate to organs such as the liver and lungs and establish colonization within the proximal tubules of the kidneys [6]. The majority of human infections are asymptomatic or subclinical; in symptomatic cases, disease manifestations typically start a week or more after exposure [6]. Symptoms typically present in a biphasic pattern: an initial leptospiremic phase followed by a potentially severe immune phase during which the majority of health complications arise [7]. Hallmarks of leptospirosis include fever, intravascular coagulopathy, endothelial damage and a proinflammatory immune response (reviewed in [7–10]). Weil’s disease is the most clinically distinguishing form of leptospirosis and includes jaundice and renal failure [6].

One leptospiral virulence strategy is production of outer surface adhesin proteins that recognize and bind components of the host plasma, extracellular matrix (ECM), and cell-surface receptors [11–49]. In pathogenic *Leptospira*, some of these proteins (e.g., secreted proteases, LenAB, LigAB, LcpA) have been shown to bind host complement components, preventing the formation of the membrane attack complex and presumably aiding in dissemination [17,18,50–53]. Pathogenic *Leptospira* also produce and secrete proteases that target host epithelial adherens junctions (AJ) proteins, but this process is not well defined [54], reviewed in [55]. Previous studies have shown that the pathogenic species *L. interrogans* disrupts host cell adherens and tight junction proteins [56]. One component of these junctions are cadherins, which maintain cell structure and cell-cell adhesion (e.g., [54,57–62]). Vascular-endothelial-cadherin (VE-cadherin) is critical for endothelial AJ maintenance and proper vascular development. Pathogenic *Leptospira* interact with and disrupt VE- and E-cadherin localization of the host cell AJs [12,13,48,49,54,56,57], although adhesion to and disruption of VE-cadherin have not been mechanistically tied [49].

Upon infection with most bacterial pathogens, the innate immune response is activated. Endothelial cells are among the first cells to interact with pathogens that enter circulation and secrete cytokines and chemokines that recruit effector cells and alter vascular permeability (reviewed in [63–65]). Production of cytokines and chemokines by endothelial cells has been well characterized *in vitro* in response to many pathogens (reviewed in [65]). These immune mediators are important for chemotaxis of leukocytes to the site(s) of infection and their adhesion to the endothelium, in the acute phase response, in stimulation of blood vessel formation, and in downstream inflammatory regulation (e.g., [66–70]). Investigation of cytokine and chemokine involvement in the endothelial response to *Leptospira* infection may reveal insights into how the pathogen manipulates host cells, including disruption of VE-cadherin localization and increasing permeability of the endothelial barrier.

The original classification of *Leptospira* was limited to a saprophytic free-living group, *Leptospira biflexa sensu lato* and a pathogenic group, *Leptospira interrogans sensu lato* [71–74]. Further analysis defined three lineages including saprophytic, intermediate, and pathogenic [75,76]. Genomic sequencing and analyses have expanded the genus *Leptospira* to include 74 different species that encompass more than 300 serovars (serological variants) [77]. Next Generation Sequencing using a multi-locus approach was necessary to further refine the taxonomy of *Leptospira* [78]. *Leptospira* is currently grouped into two clades, a saprophytic (S) and a pathogenic (P), and four subclades [77]. Saprophytic subclades are S1 and S2 and pathogenic subclades, grouped on virulence potential, were designated P1 and P2 [77]. Within the P1 subclade there are two groups: P1+ (high virulence) and P1- (low virulence) [79]. The P1 + group has altered gene expression allowing successful adaptation to the host, while limiting its ability to survive in the environment [79]. In contrast, the P1-, P2 and S groups have adapted to survive in the environment [79]. Defining differences between these subclades will further the understanding of host pathogen interactions and identification of genes important to infection and pathogenicity.

Here we compare *in vitro* and *in vivo* phenotypes of sixteen pathogenic and saprophytic strains, representative of the P1 + , P1-, P2, and S1 subclades (**Table A in** S1 File). We employed *in vitro* cell culture assays to measure *Leptospira-*endothelial cell association, disruption of AJs, and stimulation of cytokine and chemokine responses, along with an *in vivo* short-term murine model of hematogenous dissemination. Together, these data offer new insights into the phenotypic diversity of *Leptospira*, identify key virulence markers of *Leptospira*, and further refine our understanding of *Leptospira*-host interactions.

## Materials & methods

### Ethics statement

All animal studies were reviewed and approved by the Medical College of Wisconsin Institutional Animal Care and Use Committee.

### *Leptospira* strains and growth conditions

*Leptospira* strains used in this study are summarized in **Table A in** S1 File. *Leptospira* were generally grown in Ellinghausen–McCullough–Johnson–Harris (EMJH) medium [74,80,81] at 30°C, as previously described [8,12,49,56], when preparing for *in vitro* and *in vivo* experiments. When isolating bacteria from animals, HAN medium with incubation at 37°C with 5% CO_2_ was used, as recently it has been shown to enhance recovery directly from the host [82]. Bacterial concentration was determined using a Petroff-Hausser counting chamber under darkfield microscopy. Cultures were used at passage four or less from freezer stocks; pathogenic strains were used below passage eight since isolation from hamsters.

### Human dermal microvascular endothelial cells, growth, and treatments

Human dermal microvascular endothelial cells (HMEC-1) [83] were obtained from ATCC (CRL-3243) and cultured as described previously [49,56] in MCDB medium at 37°C with 5% CO_2_. Cells at passage 8 or lower were used for all experiments. Cell infection experiments were performed as previously described [12,49,56]. Briefly, HMEC-1 cells were seeded at a density of 4.8 × 10^5^ cells/well in 12-well tissue culture plates on glass coverslips and grown for 2 days until confluent. Cells were infected with 1.38 × 10^7^ late exponential phase leptospires (grown to 1–5 × 10^8^ cells/ml), equating to an MOI of ~20, and incubated for 24 hours. After incubation, cell culture medium was collected and centrifuged at 7500 x *g* for 25 minutes to remove cell debris and the majority of *Leptospira*. Resulting supernatants were aliquoted and stored at -80°C for analysis by cytokine bead array (CBA; detailed below). Cell layers on coverslips were washed with DPBS (Gibco), processed, and stained as previously described [49,56]. Cell layers were fixed with 2% paraformaldehyde and staining was performed with anti-VE-cadherin (BD Biosciences, cat. no. 610252) and AlexaFluor 488 α-mouse IgG (Invitrogen, cat. no. A11029). Coverslips were mounted with ProLong Diamond Antifade Mountant with DAPI (Invitrogen, cat. no. P36962).

### Immunofluorescence microscopy and quantification

All images were acquired on a Nikon Eclipse Ti-U inverted microscope equipped with a Hamamatsu FLASH camera and a multifluorescent Sedat Quat ET filter set (multichroic splitter, Chroma) using the 20 × Plan Apo objective lens (N.A. 0.75, Nikon, Melville, NY). NIS-Elements software (Nikon), ImageJ [84], and Microsoft Excel were used for acquisition, processing, and analysis as previously described [49]. After analysis, the binary area of VE-cadherin was subtracted from the uninfected control value set to 1 resulting in positive values for “VE-cadherin disruption.”

Representative images were selected from the four fields nearest the mean quantified disruption value. Brightness for each channel was adjusted uniformly in an unbiased manner to allow accurate comparison across sessions. For VE-cadherin, the look up tables (LUTs) were standardized based on the mean signal intensity of uninfected cells for the specific day, with the minimum set to one-third of the mean and the maximum set to three times the mean. DAPI LUTs were adjusted similarly using the average DAPI signal across all samples for the day, setting the minimum to one-third of the mean and the maximum to 1.5 times the mean. Images were merged and exported as TIFF files from Nikon Elements AR (Nikon, Melville, NY).

### Cell association assay and qPCR quantification

Bacterial association with HMEC-1 cells was quantified as previously described [49]. Briefly, 4.4 × 10^5^ cells were seeded per well in a 96-well tissue culture plate and grown to post-confluence for 3 days. Late exponential phase leptospires were pelleted at 7,500 × *g* for 20 min, resuspended in cell culture medium, and 1.5 x 10^6^ leptospires were added to each well equating to an MOI of ~20, or medium only as a control. Plates were centrifuged at 670 x *g* for 20 min, incubated for 1 hour at 37°C under 5% CO_2_, and non-associated bacteria were washed away as described [49]. DNA was harvested using the DNeasy Blood & Tissue Kit (Qiagen, cat. no. 69504), and quantitative polymerase chain reaction (qPCR) performed with primers as previously described [49,85]. Due to day-to-day variation, each set of technical replicates was averaged and normalized to *L. biflexa* sv. Patoc (*Lb*P) before being combined with the other independent experiments. Previous reports have shown that *Leptospira* internalize in cells [57,86,87], and these methods do not differentiate between adhesion and internalization, thus we use the term association to describe the interaction between bacteria and cells in these assays.

### Cytokine bead array

Preliminary experiments were performed with two LEGENDplex CBA panels (BioLegend, 741088 and 741111). A custom LEGENDplex CBA panel (BioLegend) was used to quantify CCL5 (RANTES), CXCL8 (IL-8), CXCL10 (IP-10), CCL-2 (MCP-1), IL-6, GM-CSF, G-CSF, VEGF, TNF-α, and TGF-β1. A fresh aliquot of cell culture medium harvested from HMEC-1 cell infection was thawed and CBA assay was performed according to the manufacturer’s instructions, except that the final samples were resuspended in 2% PFA diluted in wash buffer to eliminate viable bacteria. Samples were analyzed on an LSRFortessa X20 analytical cytometer. Data were processed using LEGENDplex Data Analysis Software Suite to determine analyte concentrations.

### Hematogenous dissemination infection model

Animals were housed and were fed and watered *ad libitum* according to institutional guidelines. The Medical College of Wisconsin Institutional Animal Care and Use Committee approved all animal work.

Bacteria were prepared and infections performed as previously described [85,88]. To prepare bacterial suspensions for inoculation of mice, cultures were centrifuged at 3,651 x *g* for 20 min at ambient temperature. Pellets were washed once with PBS and resuspended in PBS to a final density of 10^9^ spirochetes/ml. Data for *L. interrogans* sv. Manilae (*Li*M) were previously published [85]; the bacteria were washed and resuspended in PBS supplemented with 0.2% heat-inactivated normal C3H/HeJ mouse serum. Experiments performed with and without serum were compared for *Lb*P and *Li*M, and no significant differences were seen. To avoid any potential immune modulatory effects of serum, serum was omitted when testing the additional strains presented in this manuscript.

C3H/HeJ female mice (Jackson Laboratory, Bar Harbor, ME), aged 7 – 10 weeks, were anesthetized *via* intraperitoneal (IP) injection of ketamine-xylazine. A volume of 100 µl of bacterial suspension was inoculated intravenously via the tail vein delivering 1 x 10^8^ bacteria/mouse. Twenty minutes to 24 hours after inoculation, cardiac puncture was performed to collect blood from anesthetized mice.

The chest cavity was then opened, and mice were perfused with 0.9% sterile sodium chloride at a flow rate of 1 ml/min through the heart for 6 minutes as previously described [85,88]. Samples of lung and kidney were collected into HAN culture medium [82], and cultures were placed at 37°C and checked weekly for motile spirochetes using dark-field microscopy. Additional samples of kidney, bladder, lung, and liver were transferred to labeled 1.5 ml tubes and placed on dry ice. Tissue samples, and blood after serum separation, were stored at -80°C until DNA extraction. The frozen samples were processed to obtain DNA using the DNeasy blood and tissue kit (Qiagen, cat. no. 69504) according to manufacturer’s instructions.

### qPCR of DNA from mouse tissues

Quantification of genome copies of *Leptospira* and mouse from blood, kidney, bladder, lung, and liver was performed as previously described [85]. DNA was amplified by qPCR in 20 μl per reaction containing 5 μl of 20 ng/µl purified DNA, 10 μl of qMax Green Master Mix (Accuris Instruments), 1.8 μl ddH_2_0, and 1.6 μl each of both forward and reverse primers (5 µM stocks). Primer sets for *Leptospira 16S rRNA* were as follows: forward: 5’-TAGTG AACGGGATTAGATAC-3’, reverse: 5’-GGATGCCACAGGATTCCATACCCA-3’ [49,85,89]. Primer sets for *Mus musculus* β-*actin* are as follows: forward: ‘5-TCACCCACACTGTGCCCATCTACGA-3’, reverse: 5’-GGATGCCACAGGATT CCATACCCA-3’ [90]. A standard curve was generated and used to calculate *Leptospira 16S rRNA* gene copies from strain specific DNA and the CFX Maestro Software (Bio-Rad, Hercules, CA). Standard curves had R^2^ values of >0.99 and slopes of <-3.3 as reaction efficiency parameters. Mouse DNA at 100 ng per reaction were added to the *Leptospira* standards to replicate the conditions used to amplify DNA in samples from inoculated mice. Genomes were normalized to the inoculum to allow comparison with multiple strains performed on different days. For *L. langatensis* and *L. bandrabouensis*, the inoculum sample was lost prior to quantification, and therefore these experiments were normalized to the median inoculum of all experiments presented in this paper (median = 1.43 x 10^8^ leptospires/mL, range = 5.91 x 10^6^ to 5.57 x 10^9^ leptospires/mL). The data for *Lb*P and *Li*M were previously published [85], but analysis was updated to provide a direct comparison to the data with the other strains.

### Statistical analysis

Statistical analyses were performed using Prism (GraphPad Software). All data were analyzed by comparing strains to *Lb*P. Binding and disruption assay data were analyzed by ordinary one-way ANOVA, correcting for multiple comparisons with the Dunnett test. The hematogenous dissemination qPCR data are presented as median ± 95% confidence interval and analyzed using one-way ANOVA Kruskal-Wallis test correcting for multiple comparisons by controlling the false discovery rate using the method of Benjamini, Krieger, and Yekutieli.

### Correlation analysis

Data collected from experiments were input into Prism (GraphPad Software). Presence of virulence-associated genes was previously calculated based on known or candidate virulence factors in *L. interrogans* [79]. Data used for this analysis are in S2 File and include all the phenotypes included in this publication. A non-parametric Spearman’s rank correlation was performed. For each phenotype, strains are ranked based upon the mean or median value which is independent of error and units. A comparison between rankings across the phenotype is made to determine whether the ranking of strains is consistent across multiple phenotypes. Correlation data is output as a Spearman’s rank *r*_*s*_ and a corresponding *p* value.

## Results

### *Leptospira* spp. associate with endothelial cells and disrupt VE-cadherin in vitro

**Fig 1:**
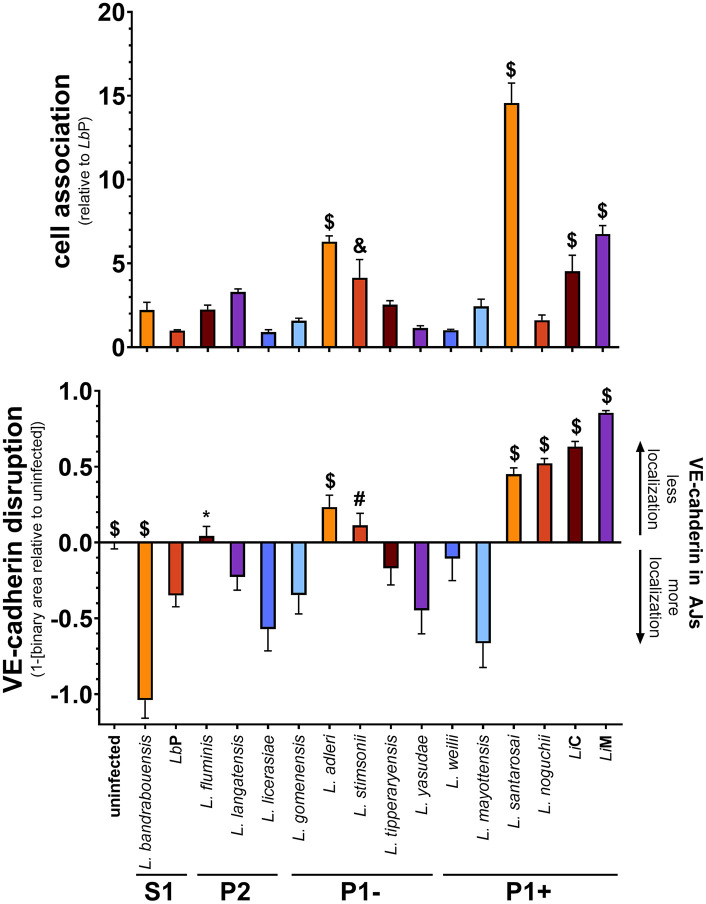
Cell association by *Leptospira* spp. correlates with VE-cadherin disruption. (top) HMEC-1 cells were grown to post-confluence, bacteria were added at an MOI of 20 and incubated for one hour. Non-associated bacteria were removed by washing and qPCR was performed to quantify associated bacteria. Strains associate with endothelial cells to varying extents, with 3/6 P1 + strains and 2/5 P1- species binding significantly more than *Lb*P. (bottom) HMEC-1 cells were grown to confluence on glass coverslips and infected at an MOI of 20 for 24 **h.** After washing to remove unbound bacteria, cells were fixed, stained for VE-cadherin, and mounted with Prolong Diamond Antifade Mountant with DAPI. Binary area was quantified as previously described [49] and subtracted from uninfected controls to define “VE-cadherin disruption”. P1 + *Leptospira* (4/6) disrupt VE-cadherin more than other clades (3/10). Spearman correlation analysis determines cell association and VE-cadherin disruption correlate with *r*_*s*_ = 0.644 and p = 0.0085. Strains are ordered based upon presence of virulence-associated genes in their genomes [79]. Mean ± SEM is plotted. Each column is compared to *LbP*. * *p* < 0.05, # *p* < 0.01, & *p* < 0.001, $ *p* < 0.0001.

We have previously shown that pathogenic *Leptospira interrogans* interacts with endothelial cells [8,12,13,49,56]. To understand these interactions in the context of the evolution of *Leptospira* and clade classification, we determined the ability of the sixteen strains representative of the clades of *Leptospira* to associate with endothelial cells and disrupt VE-cadherin. *Leptospira* association with HMEC-1 cells was performed as previously described with quantification by qPCR to determine the percentage of inoculum associated [49]. All *Leptospira* strains associate with endothelial cells to varying extents and were compared to the S1 species *Lb*P (**Fig 1**). Only members of the P1 subclade showed significantly elevated association compared with *Lb*P: three of six P1 + strains and two of five P1- species associated significantly more efficiently, suggesting this phenotype could be linked with the presence of virulence-associated genes as P1+ and P1- have similar numbers of these genes (**Table A in** S1 File). Furthermore, *Li* has been previously shown to disrupt VE-cadherin localization to AJs [49,56]. We hypothesized VE-cadherin disruption would correlate with direct bacterial-cell association, as spent medium from *L. interrogans*-infected cell layers does not affect layer integrity [8]. VE-cadherin disruption was measured by IF and quantified as previously described [49]. Only seven strains of *Leptospira* disrupted VE-cadherin compared to *Lb*P (4/6 P1 + , 2/5 P1-, and 1/3 P2) (**Figs 1 and A in** S1 File). The increased numbers in pathogenic clades suggests a correlation between clade classification and VE-cadherin disruption. One S1 strain, *L. bandrabouensis*, led to an increase in VE-cadherin localization to AJs, suggesting it stimulated cells to strengthen endothelial barriers, which requires further investigation. These two phenotypes were compared by a Spearman’s rank correlation (*r*_*s*_) and found to significantly correlate with *r*_*s*_ = 0.644 with a *p* = 0.0085. Together, these data suggest a link between endothelial cell association and VE-cadherin disruption. Overall, highly pathogenic *Leptospira* associate with and disrupt endothelial cells more than avirulent species.

### *Leptospira* spp. induce an innate immune response in endothelial cells in vitro

Following 24 hours of infection, the culture medium was collected and analyzed for cytokine production by endothelial cells. Preliminary experiments utilized two pre-defined LegendPlex (BioLegend) panels containing a total of 27 analytes to identify cytokines and chemokines that were induced by *Leptospira* when cultured with endothelial cells. A comparison between *Li*M (P1+) and *Lb*P (S1) identified seven analytes that were upregulated when HMEC-1 cells were treated with the pathogenic strain (CCL5/RANTES, CXCL8/IL-8, CXCL10/IP-10, CCL-2/MCP-1, IL-6, GM-CSF, G-CSF) (**Fig B in** S1 File).

A custom panel was created to further define the production of these analytes upon endothelial cell infection. TNF-α was included as a control, but did not rise above the limit of detection. VEGF, also known as vascular permeability factor and therefore highly relevant to our models, was not significantly altered by any species but was also included as a control. Finally, TGF-β1 was included due to its potential role in endothelial-mesenchymal transition, which due to de-differentiation of endothelia increases vascular permeability (e.g., reviewed in [91,92]).

**Fig 2:**
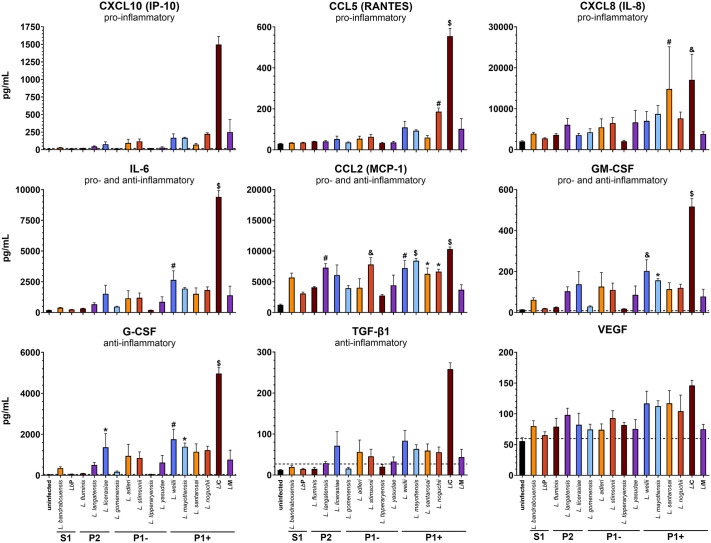
*Leptospira* spp. induce an immune response in endothelial cells. Samples were collected from cells infected for 24 hours and processed using a BioLegend LegendPlex Custom Kit. Resulting samples were analyzed on a LSRFortessa X20 analytical cytometer and processed using LEGENDplex Data Analysis Software Suite. Mean ± SEM is plotted. P1 + *Leptospira* stimulate a broad cytokine and chemokine response compared to other clades. Each column is compared to *Lb*P. Although elevated after infection with some strains, CXCL10 and TGF-β1 levels in *Lb*P-infected samples were below the limit of detection (dashed line) so statistical tests could not be performed. * *p* < 0.05, # *p* < 0.01, & *p* < 0.001, $ *p* < 0.0001.

Cytokine and chemokine production varies across the 15 pathogenic and saprophytic *Leptospira* species (**Figs 2 and B and Table A in** S1 File). Pathogenic *Leptospira* stimulate a robust cytokine and chemokine response compared to saprophytic species. Cells incubated with *L. interrogans* sv. Copenhageni (*Li*C) produce significantly higher levels of all cytokines and chemokines tested when compared to *Lb*P (**Fig 2**). *Li*M did not induce production of these cytokines or chemokines to the same level, highlighting strain differences within a given species that requires further investigation (**Fig 2**).

The chemokine CCL2 (MCP-1) functions to attract monocytes and macrophages to the site of inflammation [93]. Five of the six P1 + strains induced significantly higher levels of CCL2 (MCP-1) than did *Lb*P, while only one of the five P1- strains induced increased CCL2 (MCP-1). One P2 strain also induced increased CCL2 (MCP-1) production as compared to *Lb*P (**Fig 2**). Very few significant inductions of other cytokines or chemokines in the panel were observed with the strains tested, except for with *Li*C (**Fig 2**). CXCL8 (IL-8) is a pro-inflammatory neutrophil recruiter [70] and was significantly increased when cells were treated with *L. noguchii* (P1+) and *Li*C (P1+) (**Fig 2**). Furthermore, *L. mayottensis* (P1+), *L. weilii* (P1+), and *Li*C (P1+) induced significant levels of both G-CSF and GM-CSF (**Fig 2**). These two cytokines stimulate neutrophil development and myelomonocytic lineage cells respectively [63,94]. Together, these results indicate that pathogenic *Leptospira* species induce a stronger immune response by endothelial cells in comparison to avirulent species (**Table 1**).

**Table 1:** Phenotypes based on subclade classification.

	number of strains per clade			
*significant increases compared with* Lb*P*				
	S1	P2	P1-	P1+
**endothelial cell association**	0/2	0/3	2/5	3/6
**VE-cadherin disruption**	0/2	1/3	2/5	4/6
**CCL5 (RANTES)**	0/2	0/3	0/5	2/6
**CXCL8 (IL-8)**	0/2	0/3	0/5	2/6
**IL-6**	0/2	0/3	0/5	2/6
**CCL2 (MCP-1)**	0/2	1/3	1/5	5/6
**GM-CSF**	0/2	0/3	0/5	3/6
**G-CSF**	0/2	1/3	0/5	3/6
**VEGF**	0/2	0/3	0/5	0/6
**kidney burdens**	0/2	0/3	0/5	4/6
**bladder burdens**	0/2	0/3	1/5	4/6
**lung burdens**	0/2	0/3	0/5	0/6
**liver burdens**	0/2	0/3	2/5	1/6
**blood burdens**	0/2	0/3	2/5	4/6
***increases above LOD (*Lb*P below LOD)***				
	**S1**	**P2**	**P1-**	**P1+**
**CXCL10 (IP-10)**	1/2	3/3	4/5	6/6
**TGF-β1**	0/2	1/3	3/5	6/6
***increases above 2 times LOD (*Lb*P below LOD)***				
	**S1**	**P2**	**P1-**	**P1+**
**CXCL10 (IP-10)**	0/2	2/3	2/5	6/6
**TGF-β1**	0/2	1/3	1/5	5/6
***increases above 3 times LOD (*Lb*P below LOD)***				
	**S1**	**P2**	**P1-**	**P1+**
**CXCL10 (IP-10)**	0/2	1/3	2/5	6/6
**TGF-β1**	0/2	0/3	0/5	2/6
***increases above 4 times LOD (*Lb*P below LOD)***				
	**S1**	**P2**	**P1-**	**P1+**
**CXCL10 (IP-10)**	0/2	1/3	2/5	6/6
**TGF-β1**	0/2	0/3	0/5	1/6

LOD = limit of detection

### *Leptospira* DNA is quantifiable and living leptospires are recoverable from tissues following *in vivo* inoculation of mice with a variety strains

**Fig 3:**
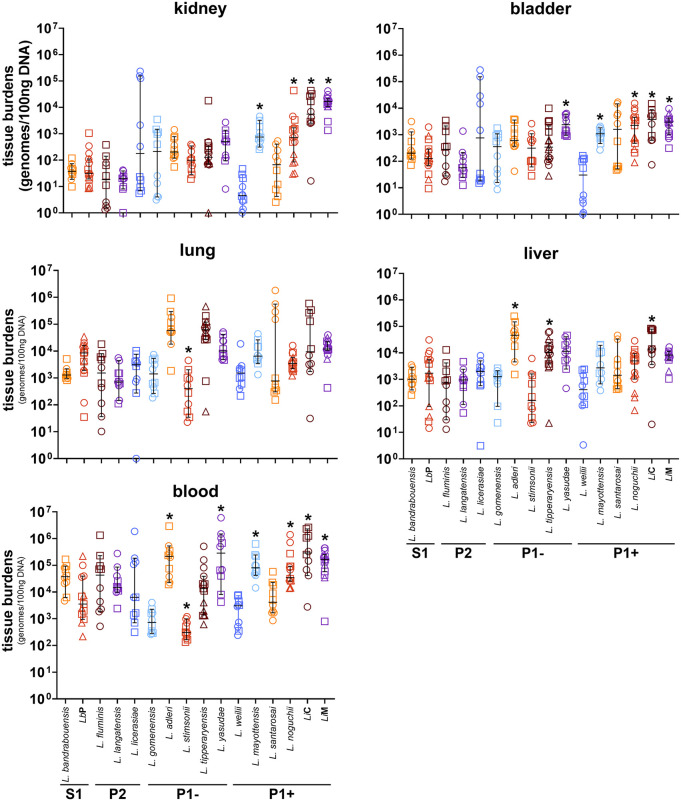
Quantification of *Leptospira* strain burdens in kidney, bladder, lung, liver and blood. C3H/HeJ mice were injected intravenously, and the bacteria were allowed to circulate for one hour. Each point represents one mouse. Circles, squares, and triangles indicate the mice belonging to biological replicates utilizing independent bacterial cultures for a total n value of 10-15 mice per strain. Data for *Lb*P and *Li*M were previously published [85], and analysis updated to provide a direct comparison to the other strains. Highly pathogenic *Leptospira* are associated with mouse tissues more than other clades of bacteria. Mean ± 95% confidence interval is plotted. * indicates a discovery based upon the two-stage linear step-up procedure of Benjamini, Krieger and Yekutieli correcting for the false discovery rate.

*Leptospira* species were evaluated in a previously described murine model of hematogenous dissemination to assess tissue tropism [85,88]. C3H/HeJ mice were inoculated intravenously with 1 x 10^8^ bacteria and bacteria were allowed to circulate for one hour before vascular perfusion was performed. Tissues were harvested for culture and qPCR analysis. All tissues from mice inoculated with the *Leptospira* strains had quantifiable bacterial burdens (**Fig 3**), but varied in comparison to the burdens of *Lb*P. Blood burdens were highest in the P1 clade, with 4/6 P1+ and 2/5 P1- strains having significantly elevated burdens compared to *Lb*P. (**Fig 3**). In the kidney, 4/6 P1 + strains had higher burdens compared to *Lb*P, whereas no P1-, P2, or S1 strains had elevated burdens. Significant burdens of 4/6 P1+ and 1/5 P1- strains were found in the bladder compared with *Lb*P. No species had significant increases in burdens in the lungs, however, *L. stimsonii* had significantly lower lung burdens than *Lb*P. In the liver, 1/6 P1 + strain and 2/5 P1- strains had significant increases compared with *Lb*P. These data indicate that higher pathogenic clade classification correlates with increased survival in the blood, and with increased tropism to the kidney and bladder. This is consistent with increased complement resistance among pathogens [79] and with the kidney being the natural site of colonization for pathogenic *Leptospira* in hosts, where bacteria are ultimately shed in the urine.

To identify viable bacteria, portions of lung and kidney were placed into HAN culture medium [82] and were monitored by dark-field microscopy for 2 weeks. Viable and recoverable *Leptospira* were identified in all mice inoculated with *L. bandrabouensis* (S1) and *L. yasudae* (P1-). In contrast, *L. fluminis* (P2), *L. adleri* (P1-), and *L. stimsonii* (P1-) had no tissue cultures positive for viable bacteria. All culture results are shown in (**Table 2**) and are grouped by subclade. These data suggest that factors beyond classical virulence determinants may contribute to tissue dissemination and tropism, although this model is a one-hour intravenous survival and dissemination model and therefore does not determine the natural ability of these strains to infect a host. Together, these data indicate that *Leptospira* species are able to survive in the host for at least one-hour post infection, consistent with our previous work showing *Lb*P survives in the host for at least 6 hours post infection (**Fig C in** S1 File and [85]).

**Table 2:** Culture-positive mouse tissue following one-hour infection.

Clade	Strain	Lung	Kidney	Mice
S1	***L. bandrabouensis***	**10/10**	**10/10**	**10/10**
S1	*L. biflexa* sv. Patoc*	**8/10**	**5/10**	**8/10**
P2	***L. fluminis***	**0/10**	**0/10**	**0/10**
P2	***L. langatensis***	**5/10**	**10/10**	**10/10**
P2	***L. licerasiae***	**5/10**	**3/10**	**5/10**
P1-	***L. gomenensis***	**6/10**	**0/10**	**6/10**
P1-	***L. adleri***	**0/10**	**0/10**	**0/10**
P1-	***L. stimsonii***	**0/10**	**0/10**	**0/10**
P1-	***L. tipperaryensis***	**1/15**	**0/15**	**1/15**
P1-	***L. yasudae***	**10/10**	**10/10**	**10/10**
P1+	***L. weilii***	**5/10**	**0/10**	**5/10**
P1+	***L. mayottensis***	**6/10**	**1/10**	**6/10**
P1+	***L. santarosai***	**5/10**	**0/10**	**5/10**
P1+	***L. noguchii***	**4/15**	**1/15**	**4/15**
P1+	***L. interrogans* sv. Copenhageni**	**10/10**	**9/10**	**10/10**
P1+	*L. interrogans* sv. Manilae*	**10/10**	**7/10**	**10/10**

*Data for *Lb*P and *Li*M were previously published [85].

### Data correlations of *in vitro* and *in vivo* results

**Fig 4:**
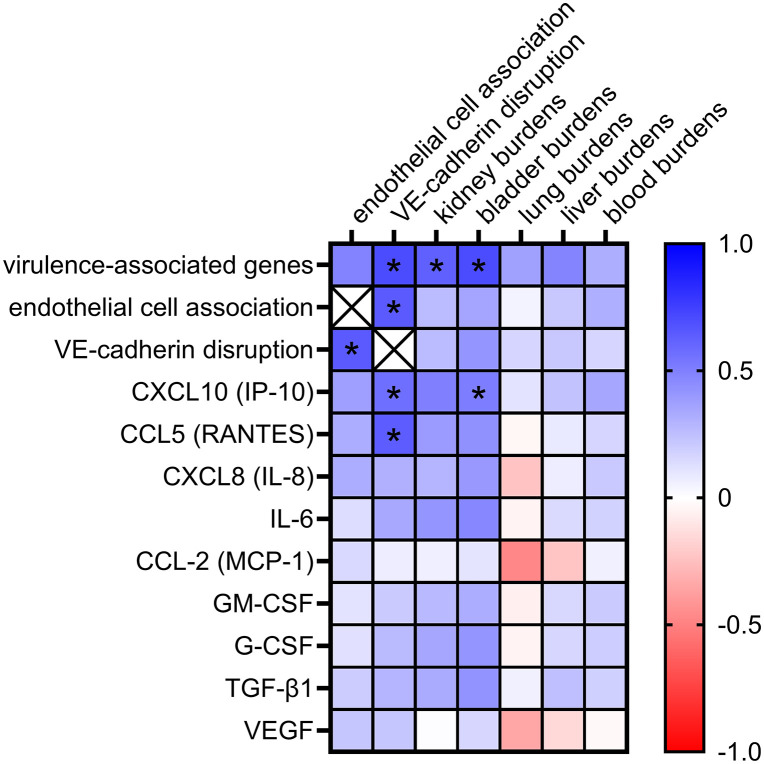
Data correlations of *in vitro* and *in vivo* results. A Spearman’s rank correlation was performed to determine whether ranking of strains for each phenotype is correlated with rankings in other phenotypes. Shown is a heat map of correlations of endothelial cell association, VE-cadherin disruption, immune factor responses, and presence of virulence-associated genes. These data identify numerous related phenotypes that may be important in *Leptospira*-host interactions. * *p* < 0.05.

A Spearman’s rank correlation between all the *in vitro* and *in vivo* phenotypes was performed to determine whether the ranking of strains for each phenotype is correlated with ranking of other phenotypes. Many of the phenotypes identified significantly correlate with each other, suggesting they are similar across strains (**Figs 4 and D in** S1 File). Endothelial cell association correlates with VE-cadherin disruption. VE-cadherin disruption correlates with increased CCL5 (RANTES) and CXCL10 (IP-10) secretion by endothelial cells, and the number of virulence-associated genes in the strains (**Fig 4**). However, CCL5 (RANTES), CXCL10 (IP-10), and the number of virulence-associated genes do not correlate with endothelial cell association, suggesting a mechanism that requires further investigation. In the hematogenous dissemination model, kidney burdens were correlated with presence of virulence-associated genes and burdens in other tissues (**Fig 4**). Bladder burdens were correlated with presence of virulence-associated genes and CXCL10 (IP-10) production (**Figs 4 and D in** S1 File). Tissue burdens all significantly correlated with each other, with the exception of no significant correlation between lung and bladder burdens (**Fig D in** S1 File). Production of cytokines and chemokines significantly correlated with each other, suggesting that strains induce a broad cytokine and chemokine response with the analytes evaluated (**Fig D in** S1 File). Together, these data identify numerous related phenotypes that may be important in *Leptospira* host interactions.

To determine whether the *in vitro* and *in vivo* phenotypes correlate with clade classification, we determined the number of strains in each clade that exhibited significantly increased phenotypes compared with *Lb*P (**Table 1**). A clear distinction between from S1 to P1 + species is seen, with more P1 + species exhibiting phenotypes than the other clades. Although CXCL10 (IP-10) and TGF-β1 levels couldn’t be analyzed for significance as *Lb*P was below the limit of detection (LOD), P1 + organisms stimulated increased production of these cytokines into the medium. Together, these data indicate that *in vitro* and *in vivo* phenotypes are markers of pathogenicity as determined by clade. Furthermore, this indicates that the known and putative virulence-associated genes identified to date are likely important contributors to host colonization and pathogenicity but are not the only considerations. Therefore, it is likely that there are unidentified virulence determinants in *Leptospira* that contribute to pathogenesis. As the analysis was performed with presence or absence of genes, and did not consider sequence variation among these genes, further mechanistic evaluation is needed and will be the subject of future work.

## Discussion

Despite the global burden of disease, the mechanisms of *Leptospira* infection and pathogenesis are not well understood. Here we provide novel insight into how our current understanding of species classification relates to phenotypes in both *in vitro* and *in vivo* experimental approaches. This work uncovers new markers of virulence that can be explored further to gain understanding of the pathogenic nature of *Leptospira*.

A set of 54 virulence associated genes was previously described [79] based upon the sequence of *Li*C and previous studies on the function of each gene. Although most genes are present within the entire P clade, with little difference in numbers present between the P1+ and P1- subclades, sequence variation does exist. This suggests that variations in homologs may be important for host adaptation and virulence. Many pathways were identified, including adhesion to host cells and the ability to escape immune clearance. Giraud-Gatineau *et al.* identified many factors acquired during the evolution of the P1 + subclade, including genes encoding uncharacterized proteins, lipoproteins, transposases, and previously identified virulence-associated genes [79]. To build on these data, we aimed to determine how strains from each clade interact with endothelial cells *in vitro* and *in vivo*, and future work will identify specific virulence-associated genes and sequences that correlate with endothelial cell interactions.

*Leptospira* species may use endothelial cell interactions to facilitate adherence to and transmigration across endothelia in multiple sites during host infection [12,41,56,87]. Our laboratory has previously shown that pathogenic *Li*C can bind VE-cadherin of human endothelial cells *in vitro*, in part due to adhesins such as LIC13411 [12,13,49]. This study reports that P1 + strains (3/6) when compared to *Lb*P (S1) demonstrate significantly greater association with HMEC-1 cells (**Fig 1 and Table 1**). Most notably, *L. santarosai* associates at least twice as efficiently to this human cell line as other P1 + strains. This suggests that *L. santarosai* may differentially express or contain sequence variation in one or more known adhesins, other virulence factors, or additional factors not identified to date (e.g., regulators of gene expression). Comparison of multiple *L. santarosai* strains and their interactions with other human endothelial cells, endothelial cells of animal origins, and other cell types will be of interest in future studies. Increased cell association is also seen in *Li*M and *Li*C, which are both well-defined virulent P1 + strains [48–50]. This phenotype may suggest one possible mechanism involved in *L. interrogans* causation of severe human disease. Two P1- species (*L. adleri* and *L. stimsonii*) rivaled *Li*M and *Li*C in the ability to associate with cells, suggesting that association of *Leptospira* to HMEC-1 cells is not a direct readout of pathogenicity in humans, as these two are not reported causes of human disease, and their ability to colonize hosts has not been firmly established. It is interesting to note that neither *L. adleri* nor *L. stimsonii* disrupted VE-cadherin localization to the extent seen with *Li*C and *Li*M. Although all tested strains displayed some level of cellular association, differences may be explained by host adaptation and specificity. Further investigation is needed to define binding to additional human and animal cell types to understand host-specificity of *Leptospira*. Additionally, a comparative analysis of sequences of known and putative virulence-associated genes encoded within a clade is needed. Defining their expression in laboratory culture conditions and in mammalian hosts will also be important to future work. Overall, endothelial cell association is a phenotype that correlates with the P1 subclade and is not observed in the P2 or S1 subclades.

Endothelial cell association of each strain correlates with *in vitro* VE-cadherin disruption, although increased association is not the sole predictor of VE-cadherin disruption (**Figs 1 and 4**). VE-cadherin disruption was evident in 4/6 P1 + strains (*Li*M, *Li*C, *L. santarosai*, and *L. noguchii*) disrupting VE-cadherin significantly more than *Lb*P (**Fig 1**). Thus, cadherin disruption may be a key marker of virulence. Our group has previously studied impacts on VE-cadherin [49,56], and others have shown similar changes with E-cadherin [54,57]. VE-cadherin disruption is also a phenotype of 2/5 P1- species, albeit to a lesser extent. This suggests a possibility for host species-, cell type-, *Leptospira* clade-, or strain-specific infection mechanisms that contribute to virulence, and identifying these mechanisms is critical to further define pathogenicity of *Leptospira*. VE-cadherin disruption correlates with increased numbers of virulence-associated *Leptospira* genes and endothelial cell association by the bacteria, but the correlation with cell association and the number of virulence-associated genes was not significant, suggesting additional unknown factors are required for these complex processes. Future work will focus on characterizing the activities of virulence-associated gene products, including presence and sequence variations, with their individual roles in cell association and cadherin disruption in multiple cell types from multiple host species.

The initial interaction of *Leptospira* with the microvascular endothelium is accompanied by the host innate immune response (e.g., [95–97]). Highly pathogenic (P1+) *Leptospira* infection of endothelial cells stimulates a broad activation of cytokine and chemokine responses (**Fig 2**). This stimulation was most prominent in *Li*C (P1+), a clinical isolate from a severe case of *Leptospirosis* [98,99]. Interestingly, a similar clinical isolate, *Li*M [100], did not induce the same level of cytokine and chemokine production, consistent with the previous observation that *Li*M did not induce a “cytokine storm” in mice even though it caused lethal infection [101]. These data also parallel a previously published difference between the two *L. interrogans* serovars. We previously found that while *Li*C binds efficiently to VE-cadherin, *Li*M does not [49]. It will be interesting to determine whether *Li*C and *Li*M have different effects on induction of innate immune responses to other cell types, and in endothelial cells of different host species. It will also be interesting to determine the spectrum of host cell receptors recognized by the two *L. interorgan* strains, which may shed light on signaling leading to different innate responses. Our data suggest that there are multiple and potentially related strain specific differences within *Leptospira* species that warrant further investigation.

*Li*C stimulated large increases in production of CXCL10 (IP-10), CCL5 (RANTES), IL-6, GM-CSF, G-CSF, and TGF-β1 in endothelial cells, despite many pathogenic strains evolving to limit immune system activation in order to escape host defenses [79]. The cytokines and chemokines elicited in response to *Li*C (P1+) infection *in vitro* raise interesting questions about this strain: are these results due to increased activation of the innate immune response or inability to block activation? How does increased cytokine and chemokine responses by endothelial cells support the pathogenicity? Overall, cytokine responses were largely related, with induction similar when correlated by species (**Fig D in** S1 File). Cytokine production correlated with the presence of virulence-associated genes and was most prominent in P1 + strains (**Fig D in** S1 File **and Table 1**). Overall, these results show that pathogenic *Leptospira* species trigger a stronger cytokine and chemokine response by endothelial cells. This response is most prominent in the highly virulent P1 + group compared with all other subclades, indicating an association between virulence and cytokine and chemokine response by endothelial cells that is absent in saprophytic strains.

One controversy in the field has been whether the widespread endothelial disruption in severe leptospirosis is due to direct actions of the bacteria, the immune response, or a combination of the two. Recent work suggested an important role for neutrophils in endothelial disruption in mice [101], but our data demonstrate that leptospires can cause endothelial damage and responses independent of immune cells. An experiment determining the kinetics of immune system activation and how this varies with *Leptospira* strain and species may provide additional insights into the endothelial response to *Leptospira*.

Interaction of *Leptospira* with the kidney and bladder endothelia in mice was associated with the P1 + group of pathogens (**Table 1**), and the presence of virulence-associated genes (**Figs 4 and D in** S1 File). In addition, P1 + organisms exhibited increased ability to survive in the blood (**Table 1**). The hematogenous dissemination model was previously used to define the role of complement during blood stream survival and dissemination of *B. burgdorferi* [88,102–105]. The data presented here are limited to one time point post-inoculation following intravenous inoculation that was optimized in previous publications [85,88], and does not address long term survival and colonization potential of the organism. In our previous work we showed that *L. biflexa* DNA was detectible for up to 24 hours in the host *via* this route of inoculation, whereas viable bacteria were only detectible for between 3 and 6 hours. In contrast, *Li*M DNA and viable bacteria were detectable and recoverable for at least 24 hours (**Fig C in** S1 File). Both *Li*M and *Li*C are known to colonize murine models for extended periods [101,106–111], reviewed in [112]. The combination of culture and qPCR data suggest that *Leptospira* DNA may survive longer in the host than do viable and recoverable leptospires*.* Overall, our data are consistent with differences in survival of strains during infection in a previous study [79], although this work was done using the hamster model of infection at different time points. Future studies will determine the time-dependent survival of species from different *Leptospira* clades in mice and other animal models of acute infection using various routes of inoculation. Given the power of mice as models for multiple infectious diseases, further development of murine models for leptospirosis will be invaluable to understanding host-pathogen interactions in this globally distributed infectious disease. It will also be important to evaluate the survival and tissue tropism of multiple pathogenic strains in other model hosts, as not all vertebrates serve as reservoirs for all pathogenic *Leptospira* species or serovars [113–115].

The data presented here provide new information to add to the established clade classification in the context of *Leptospira*-endothelial cell interactions. *Leptospira* interact with endothelial cells during initial exposure and spread to colonize the renal proximal tubules. Defining the mechanisms used by *Leptospira* to complete their life cycle is critical to developing novel preventive and therapeutic strategies targeting this potentially fatal illness. Here we have reported multiple *in vitro* and *in vivo* phenotypes that serve as markers for pathogenicity, and enable future genomic analysis to determine gene products, whether known or unknown, that may be required for successful *Leptospira* infection.

## Supporting information

S1 File Additional table and figures. **Table A. *Leptospira* strains evaluated arranged by subclade. Fig A. VE-cadherin localization is disrupted by pathogenic *Leptospira* species.** Representative images used for quantification of VE-cadherin disruption (Fig 1) are shown. Images were selected from the four fields nearest the mean quantified disruption value. Brightness for each channel was adjusted uniformly in an unbiased manner to allow accurate comparison across sessions. For VE-cadherin, LUTs were standardized based on the mean signal intensity of uninfected cells for the specific day, with the minimum set to one-third of the mean and the maximum set to three times the mean. DAPI LUTs were adjusted similarly using the average DAPI signal across all samples for the day, setting the minimum to one-third of the mean and the maximum to 1.5 times the mean. Images were merged and exported as TIFF files from Nikon Elements AR (Nikon, Melville, NY). **Fig B. Differential cytokine and chemokine responses by endothelial cells in response to P1+ and S1 *Leptospira*.** Initial experiments were performed to measure responses of endothelial cells (HMEC-1, human dermal microvascular) to P1+ and S1 *Leptospira*. LPS from *Salmonella enterica* serotype enteritidis (Sigma Aldrich, L7770) was used as a control during one replicate. These results allowed identification of cytokines and chemokines that warranted further investigation, and a custom kit was created for further experiments. Samples were collected from cells infected for 24 hours and processed using the BioLegend LegendPlex Kits #741088 (Panel 1) and #741111 (Panel 2). Resulting samples were analyzed on a LSRFortessa X20 analytical cytometer and processed using LEGENDplex Data Analysis Software Suite. Mean ± SEM is plotted. Each column is compared to every other column, unless values are below the limit of detection (dashed line). * *p* < 0.05, ** *p* < 0.01, *** *p* < 0.001, and **** *p* < 0.0001. **Fig C. Pathogenic *Leptospira* DNA and live organisms are detectible for at least 24 hours post intravenous inoculation.** *Li*M or *Lb*P were inoculated in the hematogenous dissemination model of infection, and tissues harvested at various time points. (A-E) Bacterial burdens were quantified by qPCR. Pathogenic *Leptospira* DNA is detectable in all organs at all time points, whereas non-pathogen DNA is almost eliminated by 24 hours. (F) Tissues were cultured in HAN medium to detect viable bacteria. Results are expressed as number of mice or tissue positive/total number tested. Pathogenic *Leptospira* are recoverable for at least 24 hours post-inoculation, whereas non-pathogenic bacteria are only detectible up to six hours post-inoculation. x = data not collected. Mean ± 95% confidence interval is plotted. The *Lb*P data were previously published [85], and the methods are summarized in the current manuscript. **Fig D. Expanded data correlations of *in vitro* and *in vivo* results.** A Spearman’s rank correlation was performed in GraphPad Prism comparing phenotypes identified in this study and number of virulence associated genes present in each species. Shown is a heat map of association with endothelial cells *in vitro*, VE-cadherin disruption in endothelial layers *in vitro*, immune factor responses *in vitro*, and tissue association *in vivo* correlated to virulence-associated genes representing the Spearman’s rank correlation coefficient, *r*_*s*_. * *p* < 0.05.
(PDF)

S2 File Summary of data in manuscript that is used for generation of Spearman’s rank correlation. (XLSX)
