# Supplementary material for: In vitro and in vivo endothelial interactions of Leptospira species are markers of virulence
Source: PLoS Negl Trop Dis. 2026 Jan 27;20(1):e0013939. doi: 10.1371/journal.pntd.0013939 (PMC12867331; doi:10.1371/journal.pntd.0013939)
Supplement: S1 File — Table A. Leptospira strains evaluated arranged by subclade. Fig A. VE-cadherin localization is disrupted by pathogenic Leptospira species. Representative images used for quantification of VE-cadherin disruption (Fig 1) are shown. Images were selected from the four fields nearest the mean quantified disruption value. Brightness for each channel was adjusted uniformly in an unbiased manner to allow accurate comparison across sessions. For VE-cadherin, LUTs were standardized based on the mean signal intensity of uninfected cells for the specific day, with the minimum set to one-third of the mean and the maximum set to three times the mean. DAPI LUTs were adjusted similarly using the average DAPI signal across all samples for the day, setting the minimum to one-third of the mean and the maximum to 1.5 times the mean. Images were merged and exported as TIFF files from Nikon Elements AR (Nikon, Melville, NY). Fig B. Differential cytokine and chemokine responses by endothelial cells in response to P1+ and S1 Leptospira. Initial experiments were performed to measure responses of endothelial cells (HMEC-1, human dermal microvascular) to P1+ and S1 Leptospira. LPS from Salmonella enterica serotype enteritidis (Sigma Aldrich, L7770) was used as a control during one replicate. These results allowed identification of cytokines and chemokines that warranted further investigation, and a custom kit was created for further experiments. Samples were collected from cells infected for 24 hours and processed using the BioLegend LegendPlex Kits #741088 (Panel 1) and #741111 (Panel 2). Resulting samples were analyzed on a LSRFortessa X20 analytical cytometer and processed using LEGENDplex Data Analysis Software Suite. Mean ± SEM is plotted. Each column is compared to every other column, unless values are below the limit of detection (dashed line). * p < 0.05, ** p < 0.01, *** p < 0.001, and **** p < 0.0001. Fig C. Pathogenic Leptospira DNA and live organisms are detectible for at least 24 [file pntd.0013939.s001.pdf]

S1 File. Additional Tables and Figures.

**Table A. *Leptospira* strains evaluated arranged by subclade.**				
**Clade**	**Species**	**Strain Information**	**% of virulence-associated genes***	**Reference**
S1	*L. bandrabouensis*	M10A 201601111	25.9%	(1)
S1	*L. biflexa* sv. Patoc	Patoc 1	33.3%	(2)
P2	*L. fluminis*	SCS5	64.8%	(1)
P2	*L. langatensis*	SCW18	64.8%	(1)
P2	*L. licerasiae*	VAR010	68.5%	(3)
P1-	*L. gomenensis*	KG8-B22 201800299	66.7%	(1)
P1-	*L. adleri*	FH2-B-D1 201602187	85.2%	(4)
P1-	*L. stimsonii*	SSW20 (*putramalaysiae*)	83.3%	(1, 5)
P1-	*L. tipperaryensis*	GWTS#1	85.2%	(1)
P1-	*L. yasudae*	M12A 201601115 (*dzianensis*)	83.3%	(1, 5)
P1+	*L. weilii*	LT2116	87.0%	(6)
P1+	*L. mayottensis*	200901116	87.0%	(7)
P1+	*L. santarosai*	CR2120	88.9%	(8)
P1+	*L. noguchii*	201102933	98.1%	(9)
P1+	*L. interrogans* sv. Copenhageni	Fiocruz L1-130	100%	(10, 11)
P1+	*L. interrogans* sv. Manilae	L495 UP-MMC-NIID-LP	100%	(12)

* Presence of virulence-associated genes was previously calculated based on known or candidate virulence factors in *L. interrogans* (13).

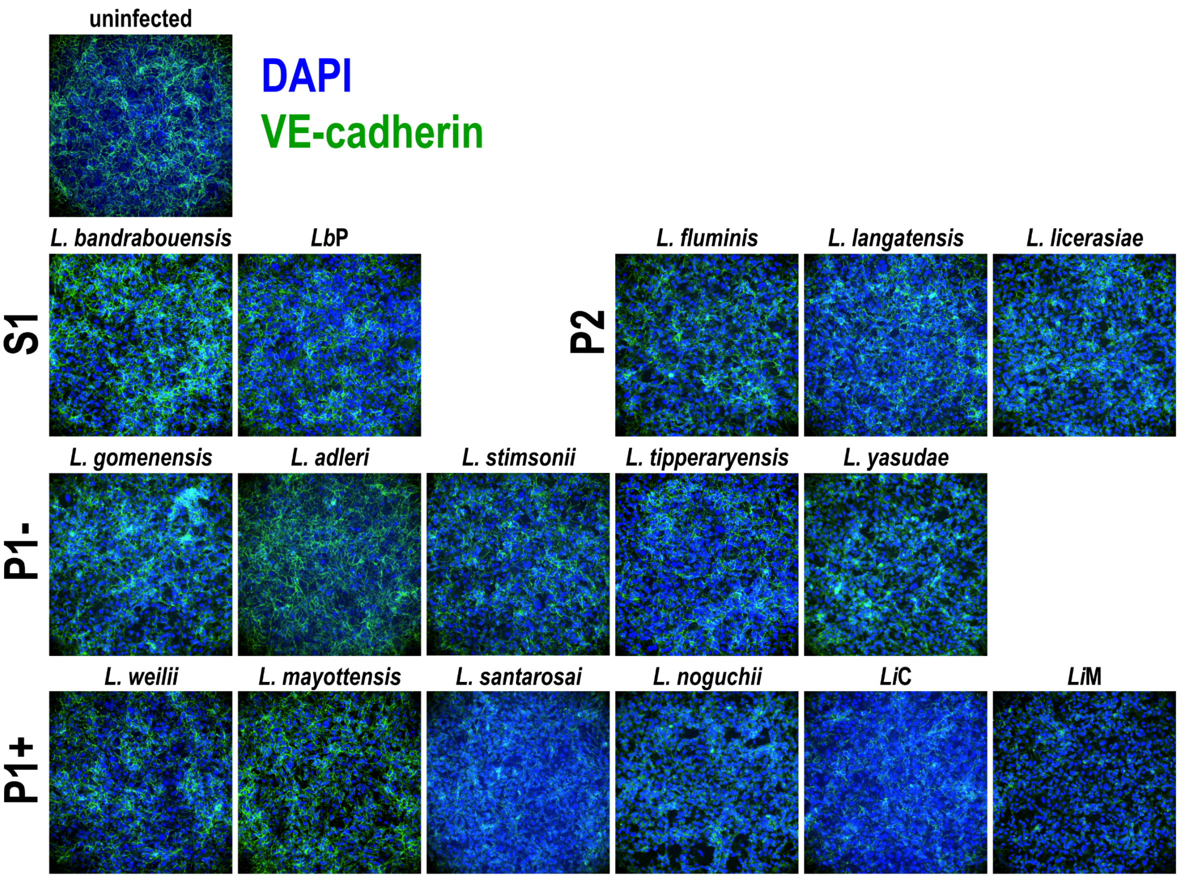

Fig A. VE-cadherin localization is disrupted by pathogenic *Leptospira* species. Representative images used for quantification of VE-cadherin disruption (Figure 1) are shown. Images were selected from the four fields nearest the mean quantified disruption value. Brightness for each channel was adjusted uniformly in an unbiased manner to allow accurate comparison across sessions. For VE-cadherin, the look up tables (LUTs) were standardized based on the mean signal intensity of uninfected cells for the specific day, with the minimum set to one-third of the mean and the maximum set to three times the mean. DAPI LUTs were adjusted similarly using the average DAPI signal across all samples for the day, setting the minimum to one-third of the mean and the maximum to 1.5 times the mean. Images were merged and exported as TIFF files from Nikon Elements AR (Nikon, Melville, NY).

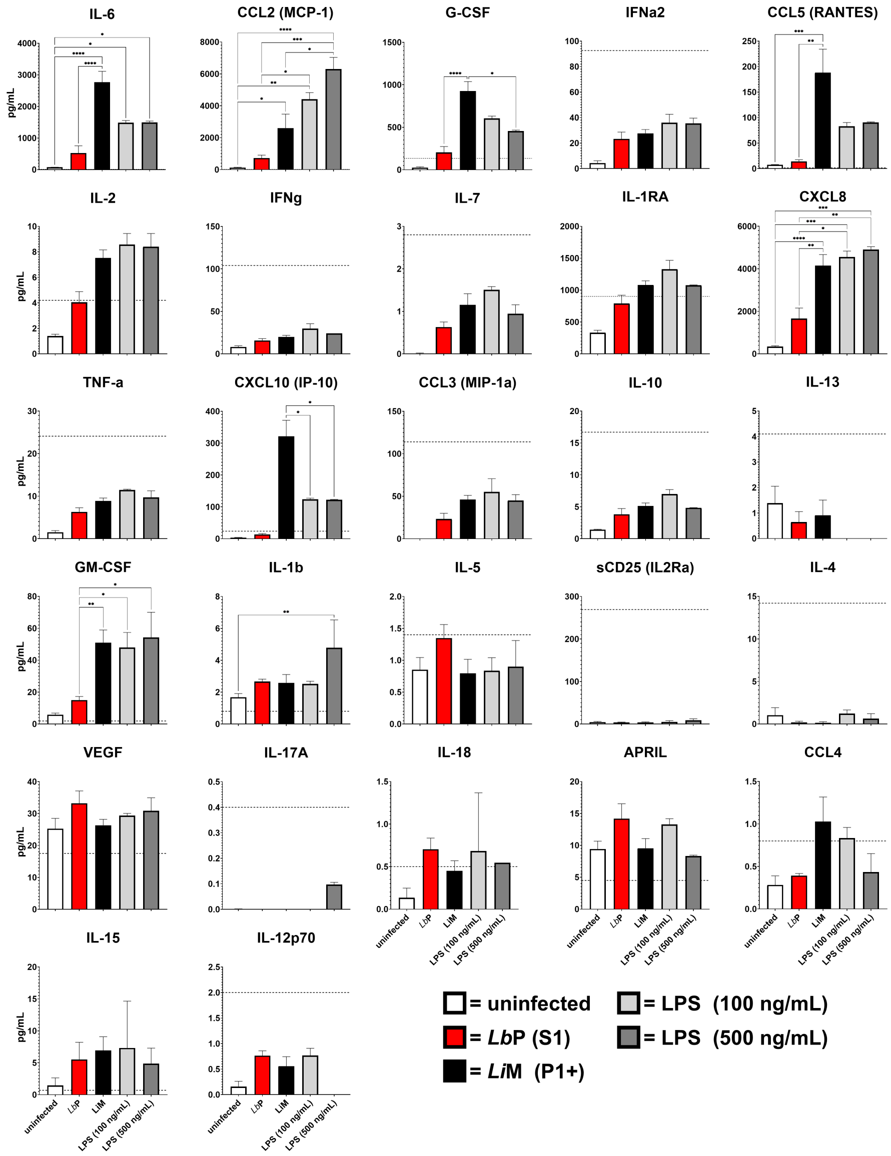

Fig B. Differential cytokine and chemokine responses by endothelial cells in response to P1+ and S1 *Leptospira*. Initial experiments were performed to measure responses of endothelial cells (HMEC-1, human dermal microvascular) to P1+ and S1 *Leptospira*. LPS from *Salmonella enterica* serotype enteritidis (Sigma Aldrich, L7770) was used as a control during one replicate. These results allowed identification of cytokines and chemokines that warranted further investigation, and a custom kit was created for further experiments. Samples were collected from cells infected for 24 hours and processed using the BioLegend LegendPlex Kits #741088 (Panel 1) and #741111 (Panel 2). Resulting samples were analyzed on a LSRFortessa X20 analytical cytometer and processed using LEGENDplex™ Data Analysis Software Suite. Mean ± SEM is plotted. Each column is compared to every other column, unless values are below the limit of detection (dashed line). * $p<0.05$, ** $p<0.01$, *** $p<0.001$, and **** $p<0.0001$.

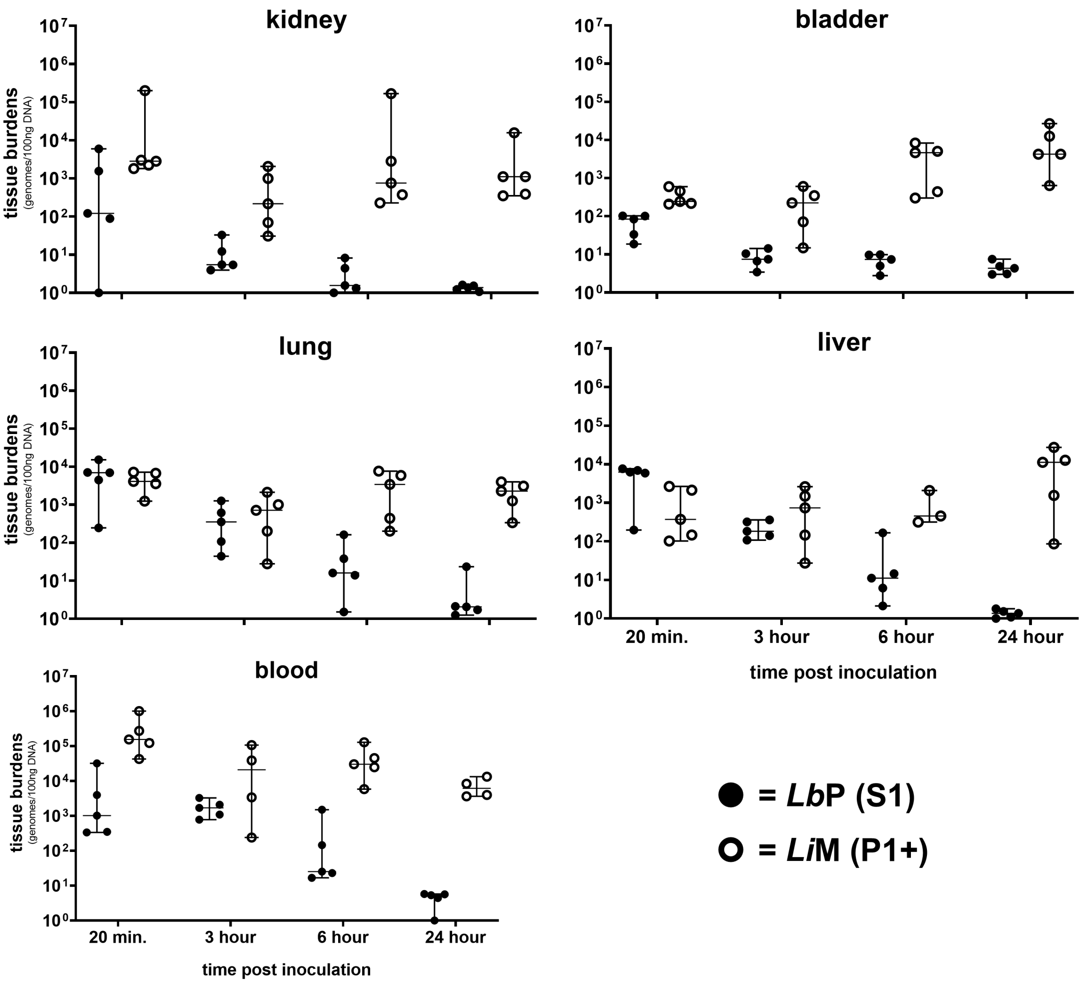

culture positive mice and tissues											
L. biflexa sv. Patoc						*L. interrogans* sv. Manilae					
**time**	**mice**	**blood**	**lung**	**liver**	**kidney**	**time**	**mice**	**blood**	**lung**	**liver**	**kidney**
**20 min.**	5/5	5/5	2/5	1/5	0/5	**20 min.**	5/5	5/5	5/5	4/5	5/5
**3 hour**	5/5	5/5	x	x	x	**3 hour**	5/5	5/5	5/5	2/5	3/5
**6 hour**	1/5	1/5	x	x	x	**6 hour**	5/5	5/5	5/5	0/5	2/5
**24 hour**	0/5	0/5	x	x	x	**24 hour**	5/5	5/5	5/5	2/5	3/5

Fig C. Pathogenic *Leptospira* DNA and live organisms are detectible for at least 24 hours post intravenous inoculation. *Li*M or *Lb*P were inoculated in the hematogenous dissemination model of infection, and tissues harvested at various time points. (A-E) Bacterial burdens were quantified by qPCR. Pathogenic *Leptospira* DNA is detectable in all organs at all time points, whereas non-pathogen DNA is almost eliminated by 24 hours. (F) Tissues were cultured in HAN medium to detect viable bacteria. Results are expressed as number of mice or tissue positive/total number tested. Pathogenic *Leptospira* are recoverable for at least 24 hours post-inoculation, whereas non-pathogenic bacteria are only detectible up to six hours post-inoculation. x = data not collected. Mean ± 95% confidence interval is plotted. The *Lb*P data were previously published (14), and the methods are summarized in the current manuscript.

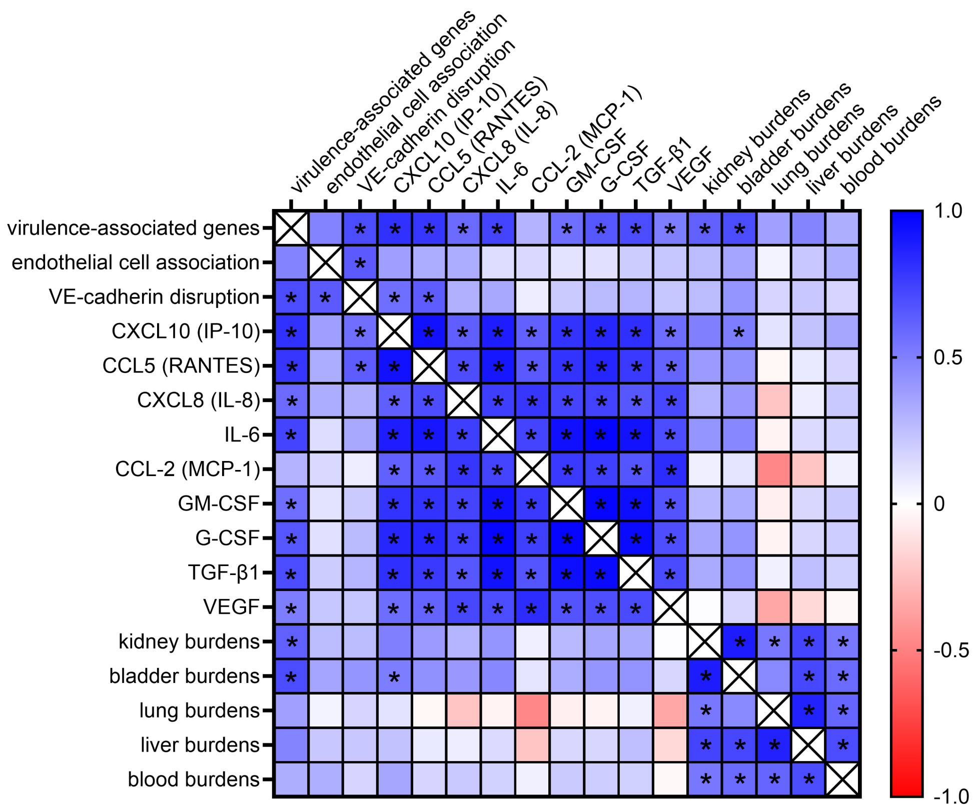

Fig D. Expanded data correlations of *in vitro* and *in vivo* results. A Spearman's rank correlation was performed in GraphPad Prism comparing phenotypes identified in this study and number of virulence associated genes present in each species. Shown is a heat map of association with endothelial cells *in vitro*, VE-cadherin disruption in endothelial layers *in vitro*, immune factor responses *in vitro*, and tissue association *in vivo* correlated to virulence-associated genes representing the Spearman's rank correlation coefficient, r_s. * $p<0.05$.

References

1. Vincent AT, Schiettekatte O, Goarant C, Neela VK, Bernet E, Thibeaux R, et al. Revisiting the taxonomy and evolution of pathogenicity of the genus *Leptospira* through the prism of genomics. PLoS Negl Trop Dis. 2019;13(5):e0007270.
2. Picardeau M, Bulach DM, Bouchier C, Zuerner RL, Zidane N, Wilson PJ, et al. Genome sequence of the saprophyte *Leptospira biflexa* provides insights into the evolution of *Leptospira* and the pathogenesis of leptospirosis. PLoS One. 2008;3(2):e1607.
3. Matthias MA, Ricaldi JN, Cespedes M, Diaz MM, Galloway RL, Saito M, et al. Human leptospirosis caused by a new, antigenically unique *Leptospira* associated with a *Rattus* species reservoir in the Peruvian Amazon. PLoS Negl Trop Dis. 2008;2(4):e213.
4. Thibeaux R, Iraola G, Ferres I, Bierque E, Girault D, Soupe-Gilbert ME, et al. Deciphering the unexplored *Leptospira* diversity from soils uncovers genomic evolution to virulence. Microb Genom. 2018;4(1).
5. Ko AI, Goarant C, Picardeau M. *Leptospira*: the dawn of the molecular genetics era for an emerging zoonotic pathogen. Nature reviews Microbiology. 2009;7(10):736–47.
6. Yasuda PH., Steigerwalt AG, Sulzer KR, Kaufmann AF, Rogers F, Brenner DJ. Deoxyribonucleic Acid Relatedness between Serogroups and Serovars in the Family Leptospiraceae with Proposals for Seven New *Leptospira* Species. Int J Syst Bacteriol. 1987;37(4).
7. Bourhy P, Collet L, Brisse S, Picardeau M. *Leptospira mayottensis* sp. nov., a pathogenic species of the genus *Leptospira* isolated from humans. Int J Syst Evol Microbiol. 2014;64(Pt 12):4061–7.
8. Chinchilla D, Nieves C, Gutierrez R, Sordoillet V, Veyrier FJ, Picardeau M. Phylogenomics of *Leptospira santarosai*, a prevalent pathogenic species in the Americas. PLoS Negl Trop Dis. 2023;17(11):e0011733.
9. Nieves C, Vincent AT, Zarantonelli L, Picardeau M, Veyrier FJ, Buschiazzo A. Horizontal transfer of the rfb cluster in *Leptospira* is a genetic determinant of serovar identity. Life Sci Alliance. 2023;6(2).
10. Nascimento AL, Ko AI, Martins EA, Monteiro-Vitorello CB, Ho PL, Haake DA, et al. Comparative genomics of two *Leptospira interrogans* serovars reveals novel insights into physiology and pathogenesis. J Bacteriol. 2004;186(7):2164–72.
11. Nascimento AL, Verjovski-Almeida S, Van Sluys MA, Monteiro-Vitorello CB, Camargo LE, Digiampietri LA, et al. Genome features of *Leptospira interrogans* serovar Copenhageni. Braz J Med Biol Res. 2004;37(4):459–77.
12. Satou K, Shimoji M, Tamotsu H, Juan A, Ashimine N, Shinzato M, et al. Complete Genome Sequences of Low-Passage Virulent and High-Passage Avirulent Variants of Pathogenic *Leptospira interrogans* Serovar Manilae Strain UP-MMC-NIID, Originally Isolated from a Patient with Severe Leptospirosis, Determined Using PacBio Single-Molecule Real-Time Technology. Genome Announc. 2015;3(4).
13. Giraud-Gatineau A, Nieves C, Harrison LB, Benaroudj N, Veyrier FJ, Picardeau M. Evolutionary insights into the emergence of virulent *Leptospira* spirochetes. PLoS Pathog. 2024;20(7):e1012161.
14. Surdel MC, Anderson PN, Hahn BL, Coburn J. Hematogenous dissemination of pathogenic and non-pathogenic *Leptospira* in a short-term murine model of infection. Front Cell Infect Microbiol. 2022;12:917962.
